# Pancreas and kidney changes in type 2 diabetes patients: the role of diffusion-weighted imaging

**DOI:** 10.3906/sag-2011-176

**Published:** 2021-06-28

**Authors:** Mehmet Hamdi ŞAHAN, Adnan ÖZDEMİR, Neşe ASAL, Yasemin Mirace KARADENİZ BİLGİLİ, Adil DOĞAN, Aşkın GÜNGÜNEŞ

**Affiliations:** 1 Department of Radiology, Faculty of Medicine, Gaziantep University, Gaziantep Turkey; 2 Department of Radiology, Faculty of Medicine, Kırıkkale University, Kırıkkale Turkey; 3 Department of Radiology, Faculty of Medicine, Kahramanmaraş Sütçü İmam University, Kahramanmaraş Turkey; 4 Department of Endocrinology and Metabolism, Faculty of Medicine, Kırıkkale University, Kırıkkale Turkey

**Keywords:** Diabetes mellitus, pancreas, kidney, magnetic resonance imaging, diffusion-weighted imaging

## Abstract

**Background/aim:**

The aim of this study was to compare renal and pancreatic apparent diffusion-coefficient (ADC) values of diabetic patients and control subjects and to examine their potential association with several diabetes-related clinical parameters.

**Materials and methods:**

A total of 80 sex- and age-matched patients were included in the study. Of them, 40 were patients with type 2 diabetes and 40 were nondiabetic participants. Abdominal diffusion-weighted MRIs of both groups were retrospectively reviewed. Diabetes-related clinical parameters were recorded.

**Results:**

The difference between the mean ADC values of the patient group and the control group was significant (p = 0.012). It was also found that the mean pancreatic ADC values of diabetic patients and the control group significantly differed (p = 0.02). Besides, there were positive correlations between the mean pancreatic ADC values and age, Hb1Ac level, treatment type, and disease duration (p < 0.05). While eGFR values positively correlated with the mean renal ADC values (p < 0.05), there were negative correlations between such values and age, serum creatinine level, and disease duration (p < 0.05).

**Conclusion:**

Renal and pancreatic ADC values of diabetic patients could potentially play a role, as markers of renal and pancreatic functions, in clinical decisions in the follow-up of such patients.

## 1. Introduction

Diabetes mellitus is considered a multisystemic disease, leading to multiple organ and system damages [1,2]. Ending with terminal kidney failure, diabetic nephropathy is among the most prevalent complications of type 2 diabetes. It is shown with glomerulosclerosis, papillary necrosis, chronic interstitial nephritis, arteriolar nephrosclerosis, various tubular lesions, and fibrosis [3,4]. In type 2 diabetes mellitus, deposits replaced within the pancreatic islet occur in patients with hyperglycemia [5,6]. It has been suggested that these islet amyloid polypeptide deposits have a considerable impact on the pathogenesis of glucose intolerance, which may be associated with progressive cell mass loss and pancreatic fibrosis [5,7]. 

Diffusion-weighted imaging (DWI) is a kind of magnetic resonance imaging (MRI) technique. It is also a technique developed based on the movements of randomly selected water molecules within the tissue; therefore, it can be used to examine tissues’ structural properties. DWI results in apparent diffusion coefficient (ADC) values, which offer considerable data about the textures examined. The ADC value, which is calculated from DWI, is a quantitative parameter. It is possible to calculate ADC values for diverse tissues by drawing a region of interest (ROI) on the map [8,9]. DWI can measure the amount of diffusion in the pancreas and kidneys and assess the damage by chronic inflammation and fibrosis [3–5,10–12]. To the best of our knowledge, the literature reserves only a few studies investigating the use of DWI for the damage to the kidneys in diabetic patients [3,4,11–14]. However, there is no study evaluating damages to both pancreas and the kidneys in such patients.

Ultimately, the present study compared renal and pancreatic ADC values of type 2 diabetes and nondiabetic control subjects and investigated their potential association with several diabetes-related clinical parameters.

## 2. Materials and methods

### 2.1. Study population and inclusion criteria

Abdominal diffusion-weighted MRIs of the patients, which had been recorded in a computer automation system from January 2015 to April 2018 in our clinic, were retrospectively reviewed. Accordingly, the study recruited a total of 80 sex- and age-matched patients; of them, 40 patients with type 2 diabetes and 40 nondiabetic control subjects satisfied the inclusion criteria of our work. The diagnosis of type 2 diabetes was considered according to the American Diabetes Association criteria [15]. Both groups’ abdominal diffusion-weighted MRI, taken for the examinations to diagnose liver and adrenal pathologies, were reviewed. Beforehand, estimated glomerular filtration rate (eGFR) values, glycated hemoglobin (HbA1c) levels, serum creatinine levels, and treatment types and disease durations within the last 1 month were retrieved from the records of type 2 diabetes patients in the endocrine polyclinic.

The primary inclusion criterion for diabetic patients was to be an adult patient with type 2 diabetes and with follow-up in our endocrine polyclinic, while the criterion for the control group was to be an adult patient without diabetes mellitus, hypertension, or heart and/or kidney diseases.

This study was carried out with regard to the Helsinki Declaration. In this context, the hospital ethics committee approved this study and granted permission to gather patients’ data for relevant examinations (Kırıkkale University Clinical Research Ethics Committee, Decision Number: 13/03, Date: May 29, 2018).

### 2.2. Exclusion criteria

The exclusion criteria for all groups were to be with pancreatic and kidney masses, to be with or to have a history of pancreatitis, to be with renal insufficiency, to have single kidney or kidney anomaly, urinary tract infections, or some other diseases, to be with liver and malignant adrenal masses affecting the kidney and pancreas anatomically and functionally, to be under the age of 18 and over 65, and to be with incomplete diffusion MRI images and data.

### 2.3. Diffusion-weighted imaging examination 

A1.5-Tesla MRI device (Philips MRI Systems, Achieva Release 3.2 Level 2013-10-21; Philips Medical Systems Nederland B.V.), using a 16-channel body torso array coil, was used to perform MRIs. DWI was done by applying diffusion-sensitive gradients with 2 different β values (β = 0 and β = 800 mm2/s) to the single-shot echo-planar sequence every 3 directions (x, y, z) in the axial plane. DWI included the following sequences: echo time (TE)/repetition time (TR) of 2335/66 ms, spectral presaturation with inversion recovery (SPIR) fat-suppressed, the field of view of 380 mm, matrix size of 128 × 128 mm, number of excitations of 2, slice thickness of 4 mm, and slice number of 15, the scan time of 28 s, slice gap of 1.2 mm, and diffusion sensitivity of 0 or 800 s/mm2. Then, the ADC maps were reconstructed through these images. Magnitude images were extracted from the MRI system to a separate workstation to calculate values and trace images.

**Figure 1:**
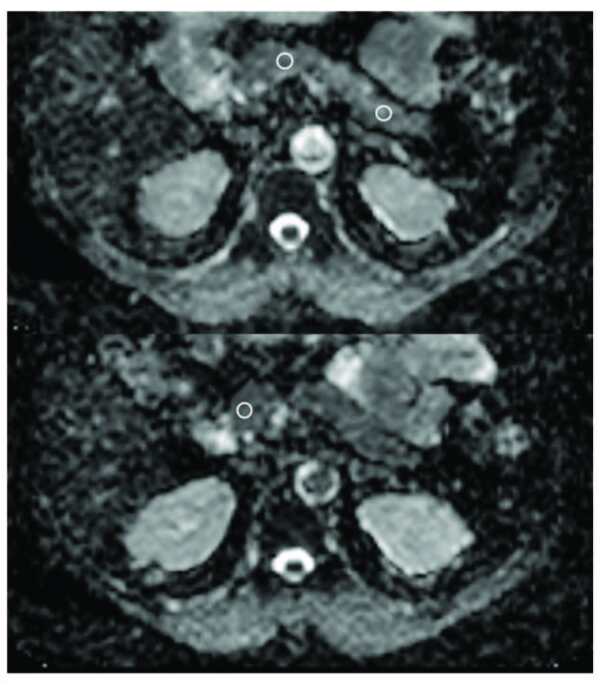
The pancreas ADC measurements of a 56-year-old female type 2 diabetes patient.

**Figure 2:**
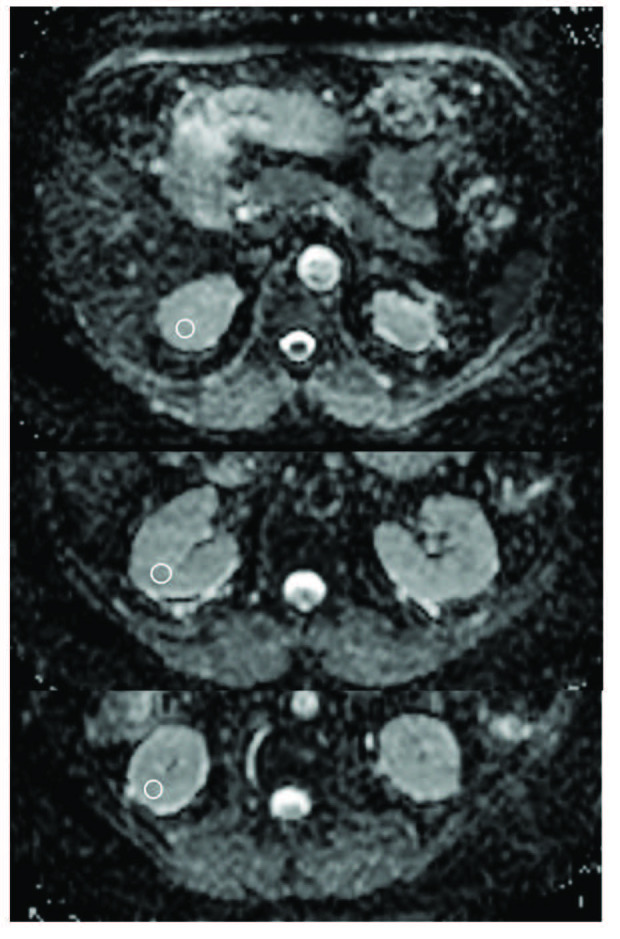
The renal ADC measurements of a 56-year-old female type 2 diabetes patient.

A single radiologist blind to clinical information (A.Ö.) examined all images on a workstation. Three ROI were drawn on the head, body, and tail of the pancreas (ovoid circles area, 30–50 mm2, Figure 1), and mean ADC values were calculated for these parts of the pancreas. After each kidney was divided into three regions (upper, mid, and lower thirds), three ROIs were drawn on each of these regions (ovoid circles area, 25–40 mm2, Figure 2). The mean ADC values were calculated for each region of the kidneys. Overall, the mean renal and pancreatic ADC values were used for statistical comparisons.

### 2.4. Statistical analysis

We used SPSS 20.0 (IBM Corp., Armonk, NY, USA) for the statistical analyses. Continuous variables were described as mean and standard deviation (SD). The independent t-test was used to detect between-group differences. Chi-square was used to compare categorical variables, such as sex, between the groups. While the Pearson product-moment correlation coefficient was calculated to reveal the relationship between numeric variables, the Spearman’s correlation rho efficient test was utilized to see the relationship between categorical variables. A p-value of 0.05 was accepted as statistically significant in all statistical analyses.

The best cut-off values for renal and pancreatic mean ADC values were identified using receiver-operating characteristic (ROC) curve analysis on the MedCalc program (version 19.5.3). Subsequently, the positive predictive value (PPV), specificity, sensitivity, and negative predictive value (NPV) were obtained. The area under the curve (AUC) was used to determine how ADC values could distinguish between the patient and control groups. 

A power analysis was performed using a 20% difference between the mean ADC values of both kidneys at an α level of 0.05 and a β level of 0.08; therefore, the minimum sample size was calculated as 78 patients. A total of 80 patients, 40 patients with type 2 diabetes, were included in the study, considering the similar comparative studies in the relevant literature [3,4,16–18].

## 3. Results

While there were 14 males and 26 females in the patient group with the mean age of 51.7 ± 9 years (range, 20–65 years), the control group was composed of 18 males and 22 females with the mean age of 51.1 ± 11 years (range, 21–65 years). The groups were congruent with each other in terms of age and sex (p > 0.05). Age, sex, HbA1c, and serum creatinine levels, treatment types, and disease durations in both groups are shown in Table 1 (treatment codes: code 1, only oral antidiabetic; code 2, oral antidiabetic + insulin; and code 3, only insulin).

**Table 1:** Clinical and demographic features of the groups.

	Type 2 diabetes patients(n = 40)	Control group(n = 40)	p values
Sex, M/F	14/26	18/22	0.494a
Age, years	51.7 ± 9 (20–65)	51.1 ± 11 (21–65)	0.781b
Hb1Ac, %	8 ± 2.2 (5.2–16)		
eGFR, mL/min	90.4 ± 24.7 (13.9–159.6)		
Creatinine, mg/dL	0.96 ± 0.64 (0.5–4.5)		
Treatment, code 1/2/3	23/6/11		
Duration of disease, years	9 ± 5.3 (0.5–20)		

Values indicate the mean ± standard deviation and range. Treatment codes, code 1: only oral antidiabetic; code 2: oral antidiabetic + insulin; code 3: only insulin; M: male, F: female. HbA1c: glycated hemoglobin, eGFR: estimated glomerular filtration rate. ap-value shows the results of chi square analysis.b

The mean renal ADC values were found to be 1977 ± 157 10–6 mm2/s in diabetic patients. In the control group, it was 2057 ± 120 10–6 mm2/s. The differences between the mean ADC values of the right/left kidney and both kidneys in the control group and the patient group were statistically significant (p = 0.012, p = 0.017, and p = 0.017, respectively) (Table 2). The mean pancreatic ADC values of the control group (1307 ± 87 10–6 mm2/s) and patient group (1390 ± 139 10–6 mm2/s) also significantly differed (p = 0.02) (Table 2).

**Table 2:** Comparison of mean ADC values of renal and pancreas between type 2 diabetes patients and control group.

	Type 2 diabetes patients(n = 40)	Control group(n = 40)	p values
Right kidney mean ADC	1932 ± 166	2008 ± 104	0.017
Left kidney mean ADC	2022 ± 159	2107 ± 152	0.017
Both kidneys’ mean ADC	1977 ± 157	2057 ± 120	0.012
Pancreas mean ADC	1390 ± 139	1307 ± 87	0.002

Values indicate the mean ± standard deviation (×10–6 mm2/s).

Meanwhile, age, Hb1Ac level, treatment type, and disease duration were significantly and positively correlated with the mean pancreatic ADC values (p < 0.05). There were also positive correlations between eGFR values and the mean ADC values of the kidneys (p < 0.05) (Table 3). Nevertheless, there were negative correlations between age, serum creatinine level, and disease duration and the mean renal ADC values (p < 0.05) (Table 3).

**Table 3:** Correlation test results in type 2 diabetes patients.

			Mean ADC values	
			Both kidneys’	Pancreas
Mean ADC values	Both kidneys’	r		–0.002
		pa		0.99
	Pancreas	r	–0.002	
		pa	0.99	
Sex		r	0.109	–0.263
		pb	0.503	0.101
Age, years		r	–0.394	0.368
		pa	0.012	0.02
Hb1Ac, %		r	0.170	0.397
		pa	0.294	0.011
eGFR, mL/min		r	0.448	0.071
		pa	0.004	0.663
Creatinine, mg/dL		r	–0.324	–0.137
		pa	0.041	0.4
Treatment, code 1/2/3		r	0.109	0.446
		pb	0.501	0.004
Duration of disease, years		r	–0.344	0.527
		pa	0.03	0.000

ADC, apparent diffusion coefficient. Code 1: male, Code 2: female. y. HbA1c: glycated hemoglobin, eGFR: estimated glomerular filtration rate. Treatment codes, code 1: only oral antidiabetic; code 2: oral antidiabetic + insulin; code 3: only insulin.ap-value shows the results of Pearson’s correlation test.bp-value shows the results of Spearman’s correlation rho efficient test.

**Figure 3:**
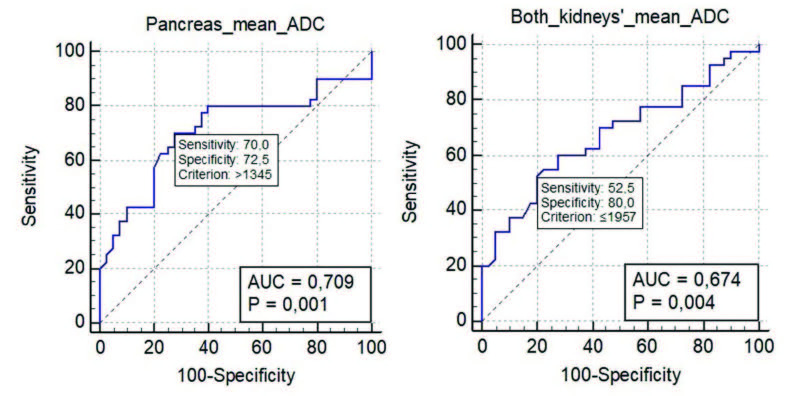
The receiver operating characteristic curves of ADC values ( 10–6 mm2/s) differentiate type 2 diabetic patients from the control group. A. For the pancreas. B. For both kidneys. The blue curve represents the receiver operating characteristic curve, and the red line represents the diagonal line used as a reference.

The ROC curve analysis revealed that the area under the curve was 0.674 (95% CI = 0.560–0.775) for the mean ADC values for both kidneys and 0.709 (95% CI = 0.597–0.806) for the mean pancreatic ADC values. The cut-off point less than 1957 10–6 mm2/s for the mean renal ADC values had a sensitivity of 52.5%, a specificity of 80%, a PPV of 72.41%, and an NPV of 62.74%. However, the cut-off point greater than 1345 10–6 mm2/s for the mean pancreas ADC values had a sensitivity of 70%, a specificity of 72.5%, a PPV of 71.79%, and an NPV of 70.73% (Figures 3A and 3B).

## 4. Discussion

We found lower renal ADC values in patients with type 2 diabetes than the control group, and there was a significant correlation between renal ADC and eGFR values. Cakmak et al. [3] reported that renal ADC values were significantly correlated with the clinical stages of diabetic nephropathy. They also determined a significant positive correlation between the mean renal ADC values and eGFR values in diabetic patients. Mrđanin et al. [4] reported that patients with diabetic nephropathy had low renal ADC values and that these values were significantly correlated with eGFR values. Inoue et al. [12] also showed that ADC values of the diabetic (n = 43) and nondiabetic (n = 76) patients suffering from chronic kidney disease were significantly correlated with eGFR values. Using diffusion tensor MRI, Lu et al. [14] discovered that the diabetic subjects had significantly lower medullary fractional anisotropy, ADC, and cortical ADC values with an eGFR < 60 compared to the control subjects. In the study of Xu et al. [19], renal ADC values were observed to be significantly lower than expected at most stages of chronic kidney disease, except stage 1, and these values were found to be negatively correlated with serum creatinine levels of the patients. Similarly, several studies in the literature previously uncovered a decrease in renal ADC values due to various acute and chronic kidney diseases and a significant correlation of eGFR values with ADC values [10–14,19,20]. This situation is likely associated with interstitial fibrosis, glomerulosclerosis, and tubular damage, as shown in the previous studies [3,11]. In addition, the present study concluded a negative correlation between the mean renal ADC values and age, serum creatinine levels, and disease durations (Table 3). 

We found a significant increase in the mean pancreatic ADC values in patients with type 2 diabetes. Such an increase in diffusion was suggested to be due to hyperglycemia in the patients and chronic inflammation and fibrosis occurring in the pancreas [5,21]. Noda et al. [22] found that in type 2 diabetes, marked acinar atrophy and pancreatic fibrosis significantly reduced islet mass and pancreatic extracellular volume was significantly higher. High extracellular volume explains the increased diffusion. Furthermore, there were significant correlations between the mean pancreatic ADC values and age, HbA1c level, disease duration, and treatment type. In general, studies on pancreatic DWI in diabetic patients are limited in the literature. Noda et al. [5] concluded that there was a potential marker between high HbA1c level and pancreatic fibrosis, which was fostered by our findings. In addition, the present study reached a correlation between patients on insulin therapy and pancreatic ADC values, which indicates an increased need for insulin due to pancreatic fibrosis.

The ROC curve analysis was also able to determine the cut-off value of the mean ADC of both kidneys (1957 10–6 mm2/s) with 52.5% sensitivity and 80% specificity, and the cut-off value of the mean pancreatic ADC (1345 10–6 mm2/s) with 70% sensitivity and 72.5% specificity. Except for the sensitivities of the renal values, sensitivities and specificities of all ADC cut-off values were calculated to be high.

There were, on the other hand, some limitations to this study. Firstly, only one reader did the ROI placement and reading, and interobserver variability could not be assessed. Secondly, ROI measurements were limited only to the renal cortex; this study did not evaluate renal medullary changes. Although multiple ROIs were placed in the cortical regions, a slight interference may have been likely with medullary areas, which is thought to have affected the results. Thirdly, no comparison was made between the nephropathy stage and ADC values. Finally, histopathologic differences were not considered in the correlation analyses. Still, the present study is strongly believed to have significant contributions to the literature. Further studies are recommended to consider and overcome the limitations mentioned above.

In conclusion, in this study, low renal ADC values in patients with type 2 diabetes were found to be significantly correlated with eGFR, serum creatinine level, age, and disease duration. Moreover, high pancreatic ADC values were revealed to be congruent with age, Hb1Ac level, disease duration, and treatment type. Renal and pancreatic ADC values of diabetic patients could potentially play a role, as markers of renal and pancreatic functions, in making clinical decisions in the follow-up of such patients.

## Disclaimer

This research did not receive any specific grant from funding agencies in the public, commercial, or not-for-profit sectors. 

## Ethical approval

This study complies with the principles of the Helsinki declaration. Ethics committee approval was obtained from Kırıkkale University Faculty of Medicine was also taken (Ethics committee decision no: 2018.05.29(13/3), date 29.05.2018).

## References

[ref1] (2004). Global prevalence of diabetes: estimates for the year 2000 and projections for 2030.

[ref2] (2017). Changes of normal appearing optic nerve head on diffusion-weighted imaging in patients with diabetic retinopathy. Clinical imaging.

[ref3] (2014). Renal diffusion-weighted imaging in diabetic nephropathy: correlation with clinical stages of disease. Diagnostic and Interventional Radiology.

[ref4] (2020). Diffusion-weighted imaging in the assessment of renal function in patients with diabetes mellitus type 2. Magnetic Resonance Materials in Physics, Biology and Medicine.

[ref5] (2016). Findings in pancreatic MRI associated with pancreatic fibrosis and HbA1c values. Journal of Magnetic Resonance Imaging.

[ref6] (1901). The relation Oe diabetes mellitus to lesions of the Pancreas. The Journal of Experimental Medicine.

[ref7] (1990). Islet amyloid polypeptide in diabetic and non-diabetic Pima Indians. Diabetologia.

[ref8] (2005). Diffusion-weighted imaging in chronic Behcet patients with and without neurological findings. Neuroradiology.

[ref9] (1991). MR imaging of anisotropically restricted diffusion of water in the nervous system: technical, anatomic, and pathologic considerations. Journal of Computer Assisted Tomography.

[ref10] (2007). Diffusion-weighted magnetic resonance imaging in the evaluation of renal function: a preliminary study. La Radiologia Medica.

[ref11] (2012). Magnetic resonance diffusion tensor imaging for evaluation of histopathological changes in a rat model of diabetic nephropathy. Investigative Radiology.

[ref12] (2011). Noninvasive evaluation of kidney hypoxia and fibrosis using magnetic resonance imaging. Journal of the American Society of Nephrology.

[ref13] (2003). Renal diffusion and BOLD MRI in experimental diabetic nephropathy. Journal of Magnetic Resonance Imaging: An Official Journal of the International Society for Magnetic Resonance in Medicine.

[ref14] (2011). Use of diffusion tensor MRI to identify early changes in diabetic nephropathy. American Journal of Nephrology.

[ref15] (2017). American diabetes association standards of medical care in diabetes 2017. Diabetes Care.

[ref16] (2018). Intravoxel incoherent motion (IVIM) at 3.0 T: evaluation of early renal function changes in type 2 diabetic patients. Abdominal Radiology.

[ref17] (2016). Detection of chronic brain damage by diffusion-weighted imaging with multiple b values in patients with type 2 diabetes. Medicine.

[ref18] (2018). Vitreous humor diffusion changes in Behçet’s disease and multiple sclerosis. Current Medical Imaging.

[ref19] (2007). Relationship between the renal apparent diffusion coefficient and glomerular filtration rate: preliminary experience. Journal of Magnetic Resonance Imaging: An Official Journal of the International Society for Magnetic Resonance in Medicine.

[ref20] (1999). Measurement of the apparent diffusion coefficient in diffuse renal disease by diffusion‐weighted echo‐planar MR imaging. Journal of Magnetic Resonance Imaging: An Official Journal of the International Society for Magnetic Resonance in Medicine.

[ref21] (1988). Islet amyloid, increased A-cells, reduced B-cells and exocrine fibrosis: quantitative changes in the pancreas in type 2 diabetes. Diabetes research (Edinburgh.

[ref22] (2020). Pancreatic extracellular volume fraction using T1 mapping in patients with impaired glucose intolerance. Abdominal Radiology.

